# Circulating tumor cells correlating with Ki-67 predicts the prognosis of bladder cancer patients

**DOI:** 10.1007/s11255-022-03406-y

**Published:** 2022-11-05

**Authors:** Jie Liu, Cailing Ma, Xiaohang Li, Anan Li, Zhiyong Wang

**Affiliations:** 1 Department of Urology, The Affiliated Hospital of Chengde Medical University, No. 36 Nanyingzi Street Chengde, Heibei, 067000 China; 2 Department of Radiology, The Affiliated Hospital of Chengde Medical University, No. 36 Nanyingzi Street Chengde, Heibei, 067000 China

**Keywords:** Bladder cancer, Circulating tumor cells, Ki-67, Prognosis

## Abstract

**Purpose:**

To investigate the value of circulating tumor cells (CTCs) and Ki-67 in predicting the prognosis of bladder cancer. Here, we tested CTC counts and Ki-67 levels to assess patient prognosis.

**Methods:**

84 patients with bladder cancer who underwent surgery were included in this study. Peripheral blood CTCs were detected by SE-iFISH technology before and after surgery, and Ki-67 levels were analyzed by immunohistochemistry. The association between CTCs and Ki-67 and the combination of the two was analyzed to predict the prognosis of patients.

**Results:**

76 out of 84 patients (90.5%) were positive. ROC curve analysis showed that preoperative and postoperative CTC counts = 4 and 2 were the best thresholds for predicting patient recurrence or death. In multivariate analysis, high postoperative CTC count (≥ 2) (*P* < 0.001) and Ki-67 high expression (≥ 15%) (*P* < 0.001) were independent poor prognostic factors for PFS in bladder cancer patients. In addition, the study found that Ki-67 levels were positively correlated with high postoperative CTC counts, Bladder cancer patients with Ki-67 high expression and high postoperative CTC counts were associated with extremely poor progression-free survival.

**Conclusion:**

Ki-67 high expression is associated with high postoperative CTC counts, both of which predict poor prognosis in bladder cancer patients.

## Introduction

Bladder cancer is one of the most common malignancies of the urinary tract. Globally, bladder cancer has the 13th highest mortality rate [1]. The annual mortality rates for men and women are 3.3 and 0.86 per 100,000, respectively [2]. There is no doubt that transurethral resection of bladder tumor (TURBT) is currently one of the main options for treating patients with non-muscle-invasive bladder cancer (NMIBC).

However, the efficacy of surgery is limited by the biological characteristics of bladder cancer, such as the high recurrence rate and rapid progression, which is accompanied by an increase in the number of recurrences after surgery and a corresponding increase in the malignancy of the lesion. For example, there is an increased probability of muscle invasion or metastasis of the tumour occurring [3]. Therefore, identifying effective predictors of tumour recurrence or metastasis in postoperative patients would have some clinical application. Currently, tumour staging and tumour grading are two key prognostic factors that clinicians rely on when attempting to individualise and provide effective treatment to patients with bladder cancer. Imaging techniques (e.g. ultrasound, CT and MRI) can provide initial information about tumour tissue invasion, however, it can be hampered by subjective judgement and lack of accuracy [4]. The acknowledged gold standard for tumour staging and grading is histological biopsy, but this is usually performed postoperatively, which is not conducive to long-term follow-up of patients after surgery. There is, therefore, a need for a timely and easily accessible complementary technique to reflect risk stratification in patients with bladder cancer.

Circulating tumor cells (CTCs) and CTCs karyotype have been used to assess the risk of tumor recurrence and metastasis [5]. CTCs belong to a subset of tumor cells, which refer to cells that are shed from tumor lesions and enter the peripheral blood circulation under the condition of spontaneous or therapeutic operation. It is currently recognized that the existence of CTCs is one of the main reasons for tumor recurrence or metastasis. Studies have demonstrated the prognostic value of CTCs testing in breast, lung, and prostate cancers [6–8]. Abnormally alternating (decreased or increased) chromosomes in cells are well-recognized markers of malignancy, studies have found that aneuploid karyotypes in CTCs can help explore cancer prognosis and treatment efficacy [9, 10]. In addition, Ki-67, a nuclear protein associated with ribosomal RNA transcription, is a marker of cell proliferation that is clearly correlated with tumor malignancy. Studies have shown the prognostic importance of Ki-67 in the recurrence and metastasis of bladder tumors [11]. In this study, we used SE-iFISH (subtraction enrichment-immunofluorescence in situ hybridization, SE-iFISH) technology to enrich and identify CTCs and CTCs karyotype, focusing on the important risk factors affecting the prognosis of patients, In particular, this study explored for the first time the significance of postoperative CTC counts and Ki-67 levels in predicting progression-free survival of patients. It provides accurate prognostic information for predicting bladder cancer patients.

## Materials and methods

### Research subjects

The study was conducted on 84 patients with bladder cancer who were first seen at the Affiliated Hospital of Chengde Medical University from 2018 to 2019. The inclusion criteria were as follows: (1) age between 18 and 90 years; (2) histopathology was the gold standard for the diagnosis and recurrence or metastasis of patients before and after surgery. All histopathological types were Urinary Tract Transitional Cell Carcinoma; (3) the patients did not receive any adjuvant anti-cancer therapy, such as surgery, radio chemotherapy, or targeted therapy before enrollment; (4) the patient has complete basic information, histopathology, and CTCs test results. The exclusion criteria were as follows: (1) those with severe heart, brain, liver, kidney, and other important organ dysfunction who cannot tolerate surgery; (2) patients with a history of other malignant tumors; (3) receive relevant anti-cancer treatments such as surgery, radiotherapy, chemotherapy, neoadjuvant chemotherapy or immunotherapy before collecting blood samples; (4) hemolysis, coagulation or the sample volume is less than 7.5 ml in the CTCs detection sample. A total of 84 bladder cancer patients were included in this study, including 44 males and 40 females. The mean age was 64.7 ± 9.9 (38–87) years old, The TNM staging of bladder cancer patients is based on the 8th edition of the Cancer Staging System of the International Union Against Cancer [12], Included 20 cases in T_a-1_ stage, 64 cases in T_2-4_ stage, 16 patients with lymph node metastasis and 68 patients without lymph node metastasis.

7.5 ml of peripheral venous blood was collected from patients 1 week before surgery, and samples were collected again 1 week after surgery. The collected samples were stored in BD anticoagulation tubes. The time when the blood was drawn and the time when the test was started was controlled within 48 h. The study was approved by the Ethics Committee of the Affiliated Hospital of Chengde Medical University [CYFYLL2022167]. An exemption of informed consent was obtained because of the retrospective nature of the study.

### Analysis of CTCs and CTCs karyotype characteristics captured by SE-iFISH platform

The experimental procedure was similar to the results of published studies [13, 14], and CTCs were enriched according to the Negative Enrichment Kit instructions (Cytelligen, San Diego, CA, USA). In Brief, the blood samples were centrifuged in a centrifuge at 23 °C 1956 rpm for 8 min, Removal of plasma yields rich leukocytes and tumor cells, Centrifuge the supernatant in a centrifuge at 1467 rpm for 8 min, after adding 200 µL of magnetic bead buffer to immunomagnetic beads and removing leukocytes under the action of the magnetic field of the magnetic rack. After the cell suspension rich in CTCs was obtained, it was centrifuged at 1467 rpm for 8 min in a centrifuge. Then, iFISH staining was performed to identify, Cell suspensions were taken and added to the chromosome 8 attachment probe (CEP8) (orange) (Cytelligen, SanDiego, CA, USA). Denature at 76 °C for 10 min, hybridize at 37 °C for 4 h. Then, add CD31 (green) monoclonal antibody, CD45 (red) monoclonal antibody, and finally add DAPI (blue) nuclear staining solution, CTCs were identified under a fluorescence microscope, and the karyotype of CTCs was judged by analyzing the fluorescence signal of CEP8. CTCs were defined as standard: DAPI + , CD45− , CD31− , CEP8 +  ≥ 3 ploidy.

**Fig. 1:**
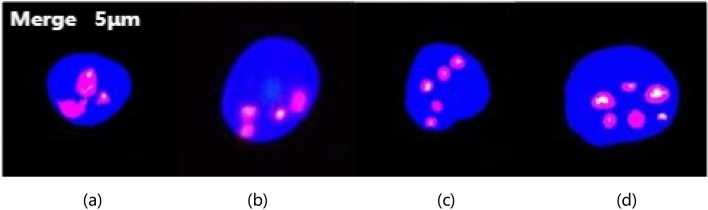
Images of CTCs karyotype in bladder cancer patients by subtraction enrichment and immunostaining-fluorescence in situ hybridization (SE-iFISH) under fluorescence microscope (× 400). **a** Triploid CTCs; **b** Tetraploid CTCs; **c** Pentaploid CTCs; **d** Multiploid CTCs

The karyotype is defined according to the amount of orange fluorescent signal (CEP8) in the nucleus of CTCs. Divided into triploid, tetraploid, pentaploid, and multiploid CTCs in turn (Fig. 1), We grouped the patients according to the number of triploid CTCs in the test results, Patients with triploid CTC count ≥ 60% were classified as triploid group and patients with < 60% were classified as non-triploid group [15].

### Ki-67 detection

The expression of Ki-67 in pathological sections was detected by pathological immunohistochemistry. SP kit, rabbit anti-human Ki-67 monoclonal antibody, and DAB chromogen were purchased from Beijing Zhongsun Jinqiao Biotechnology Co. The experimental procedures were carried out in strict accordance with the reagent instructions [16]. Ki-67 was mainly stained with nuclei in bladder cancer tissues, with brownish-yellow or tan granules. A double-blind method was used, with 2 pathologists reading the films separately. 5 different fields of view (× 400) were randomly selected under the microscope, and 200 cells were counted in each field of view. Calculate the percentage of positive fine (number of positive cells/total cells × 100%). According to previous literature, the rate of Ki-67 positive cells after staining was used to classify the Ki-67 low and high expression groups at 15% [17].

### Follow-up

All patients were checked every 3 months within 2 years after surgery, and every 6 months after the 3rd year. Review by evaluating cystoscopy, urinary ultrasound, urinary CT, and lung CT. When tumor recurrence or metastasis is suspected, cystoscopy + pathological biopsy should be performed immediately to confirm. The endpoint event for this trial was either recurrence, metastasis, or death, defined as progression-free survival (PFS). It is defined as the time interval between the first collection of a blood sample and the patient's recurrence, metastasis, or death after surgery. The follow-up period ends in June 2022.

### Statistical analysis

Analysis was performed using GraphPad Prism 7.0 (CA, USA) and SPSS 26.0 (IBM Corp) statistical software. Measurement data are described by mean ± standard deviation (X ± S) and counts were expressed as frequencies and percentages. Changes in CTCs before and after surgery were analyzed by paired sample *t* test. Categorical variables were analyzed using Chi-square test; a receiver operating characteristic curve (ROC) was plotted and a cut-off value for the predicted prognosis of CTCs was calculated when the maximum Jorden index (sensitivity + specificity − 1) was taken; Survival curves were drawn by Kaplan–Meier and Log-rank test was used to compare the differences of survival curves; Analysis by Cox proportional hazards regression model to identify independent risk factors for PFS in patients; Spearson correlation analysis was used to determine the correlation between Ki-67 levels and CTC counts. *P* < 0.05 was considered statistically significant.

## Results

### Clinical characteristics of patients

Basic information of patients with bladder cancer. The basic information of the 84 bladder cancer patients is shown in Table 1. Among them, 41 patients (48.8%) with tumor diameter ≥ 3 cm and 43 patients (51.2%) with tumor diameter < 3 cm, respectively. There were 25 (29.8%) patients with low-grade histological grades and 59 (70.2%) patients with high-grade histology. After a median follow-up of 29 (3–54) months, 27 patients (32.1%) experienced tumor recurrence or death, and the organs involved in recurrence or metastasis included bladder in 18 patients (66.7%), lung in 3 patients (11.1%), liver in 1 case (3.7%), 5 cases (18.5%) died. Immunohistochemical analysis of the degree of Ki-67 expression in pathological tissues was performed using Ki-67 = 15% as the grouping level. 28 (33.3%) of the 84 patients had Ki-67 ≥ 15% as the high expression group and 56 (66.7%) had Ki-67 < 15% as the low expression group.

**Table 1:** Basic information of enrolled bladder cancer patients

Characteristics	Groups	Total (*N* = 84)	
		Number (*N*)	Percentage (%)
Gender	Male	44	52.4
	Female	40	47.6
Age	≥ 65	33	39.3
	< 65	51	60.7
Tumor diameter	≥ 3	41	48.8
	< 3	43	51.2
T staging	T_2-4_	64	76.2
	T_a-1_	20	23.8
Lymph node metastasis	Yes	16	19.0
	No	68	81.0
Ki-67 expression	High	28	33.3
	Low	56	66.7

### SE-iFISH detection results

**Fig. 2:**
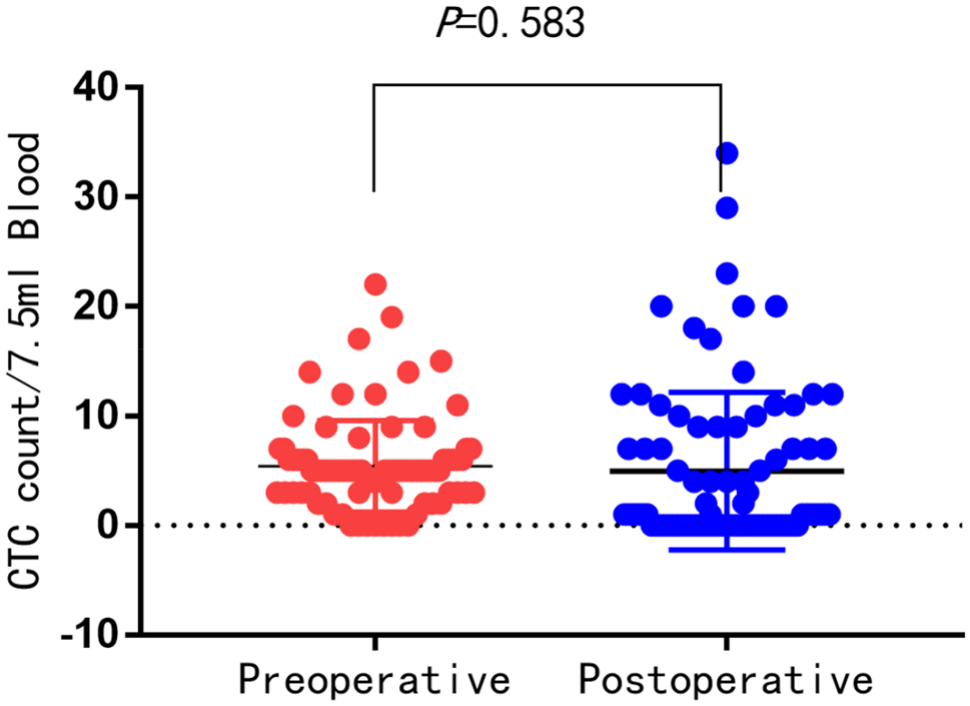
Distribution of preoperative and postoperative CTC counts in bladder cancer patients

Patients were tested twice before and after surgery using the SE-iFISH technique, the result is shown in Fig. 1. Among them, CTCs were detected in 76 (90.5%) of 84 patients before surgery, with a mean CTC count of 5.4 ± 4.2 (range, 0–22), and in 51 patients (60.7%) after surgery, with an average of 51 (60.7%). CTC counts were 5.0 ± 7.2 (range 0–34). There was no significant difference in the changes of CTC counts before and after surgery (Fig. 2, *P* = 0.573).

A total of 456 CTCs were tested before surgery, of which 150 (32.90%) were triploid (CEP = 3), 128 (28.06%) were tetraploid (CEP = 4) and 178 (39.04%) were polyploid (CEP ≥ 5). 418 CTCs were tested after surgery. 188 (45.0%) of these were triploid (CEP = 3), 97 (23.20%) were tetraploid (CEP = 4) and 133 (31.80%) were polyploid (CEP ≥ 5). According to the above definition criteria, there were 47 patients with triploid karyotypes and 37 patients with non-triploid karyotypes before surgery, 22 patients with triploid karyotypes, and 62 patients with non-triploid karyotypes after CTCs surgery.

### The analysis of the relationship between CTC count and prognosis

**Fig. 3:**
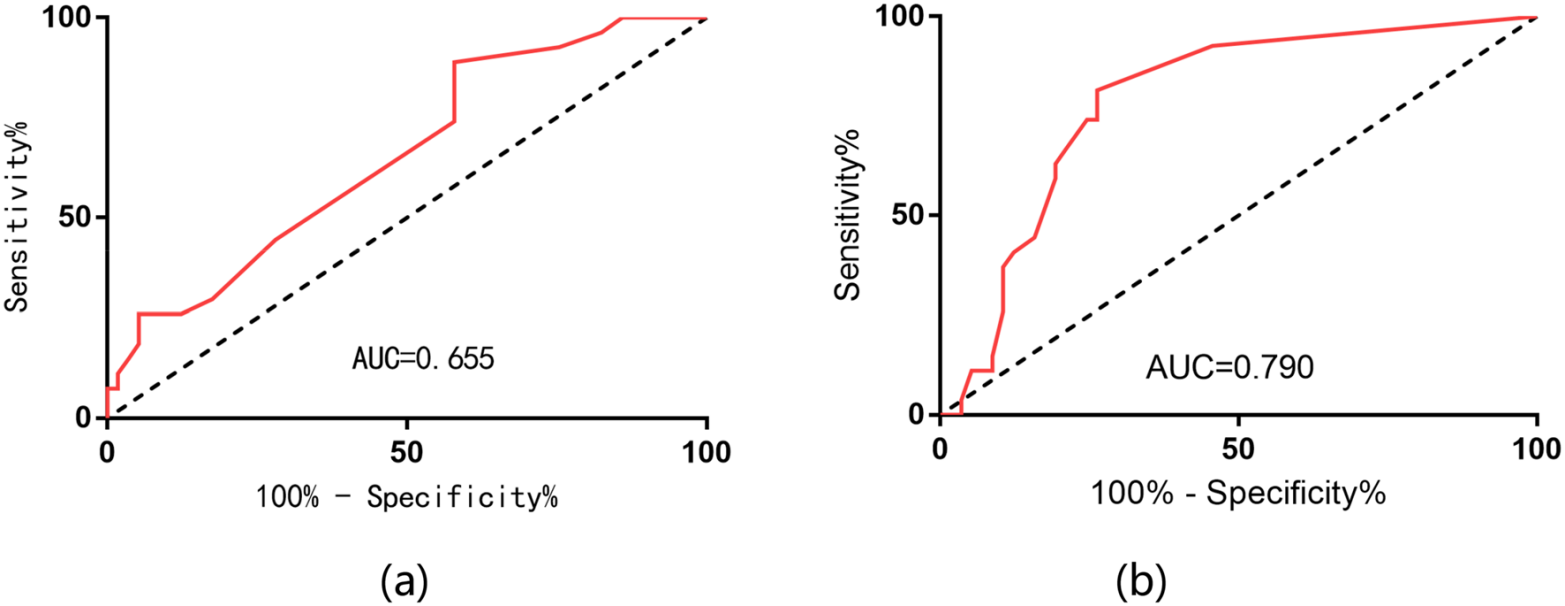
Correlation between CTC count and PFS in bladder cancer patients. **a** ROC curve analysis for the range of preoperative CTC cutoff values for PFS; **b** ROC curve analysis for the range of postoperative CTC cutoff values for PFS

**Fig. 4:**
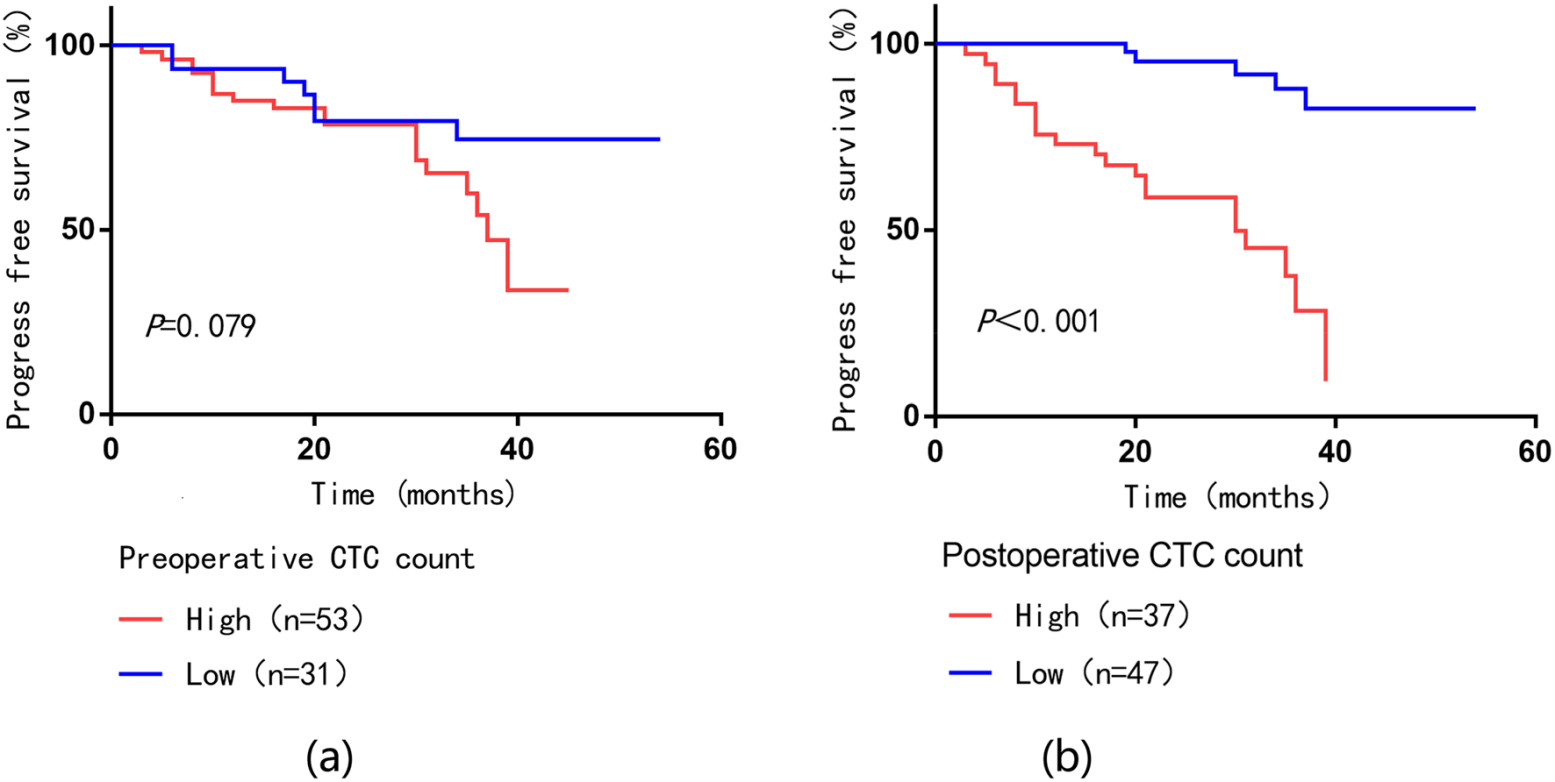
Kaplan–Meier curve analysis of the survival relationship between PFS and CTC count. **a** Preoperative CTC count; **b** Postoperative CTC count

We used ROC curves to determine whether preoperative CTC counts were predictive of recurrence or death in bladder cancer patients, and the results are shown in Fig. 3a. ROC curve analysis showed that preoperative CTC count = 4 (AUC = 0.655; 95% CI 0.534–0.777; *P* = 0.022) was the best cut-off value for predicting recurrence or death. The clinical data of patients with CTC count cutoff value = 4 are summarized in Table 2. Next, the patient’s prognosis was assessed based on the CTC count. As shown in Fig. 3a, Kaplan–Meier analysis showed that 31 patients (36.9%) with low CTC counts (< 4/7.5 ml) had lower recurrence or mortality (22.6% vs. 37.7%) compared to 53 patients (63.1%) with high preoperative CTC counts (≥ 4/7.5 ml), the difference was not statistically significant (Fig. 4a,  *P* = 0.079).

**Table 2:** The clinical characteristics of patients with bladder cancer in relation to preoperative CTCs and postoperative CTCs

Variables	Groups	Preoperative CTCs		*P* value	Postoperative CTCs		*P* value
		CTC ≥ 4	CTC < 4		CTC ≥ 2	CTC < 2	
Gender	Male	30	14	0.311	17	27	0.295
	Female	23	17		20	20	
Age	≥ 65	30	21	0.313	21	30	0.510
	< 65	23	14		16	17	
Tumor diameter	≥ 3	23	18	0.194	15	26	0.179
	< 3	30	13		22	21	
T staging	T_2-4_	43	21	0.164	34	30	0.003
	T_a-1_	10	10		3	17	
Lymph node metastasis	Yes	10	6	0.956	10	6	0.098
	No	43	25		27	41	
Ki-67 expression	High	20	8	0.263	21	7	< 0.001
	Low	33	23		16	40

ROC curve analysis in patients with postoperative CTC detection, the results are shown in Fig. 3b. Postoperative CTC count = 2(AUC = 0.790; 95% CI 0.689–0.890; *P* < 0.001) was the best cut-off value for predicting recurrence or death. Table 2 analyzes the clinical data of patients stratified by postoperative CTC count = 2. Survival analysis showed that patients with high postoperative CTC counts (≥ 2/7.5 ml) had significantly higher recurrence or mortality than those with low CTC counts (< 2/7.5 ml) (59.5% vs. 10.6%, *P* < 0.001). These results showed that patients with high postoperative CTC counts (≥ 2/7.5 m) were more prone to poor prognosis (Fig. 4b, P < 0.001). This suggests that both our preoperative and postoperative CTC counts have some predictive value for the prognosis of patients with bladder cancer, but the value of postoperative CTC is more pronounced.

### Postoperative CTC count and Ki-67 level are predictors of postoperative tumor PFS for bladder cancer

**Fig. 5:**
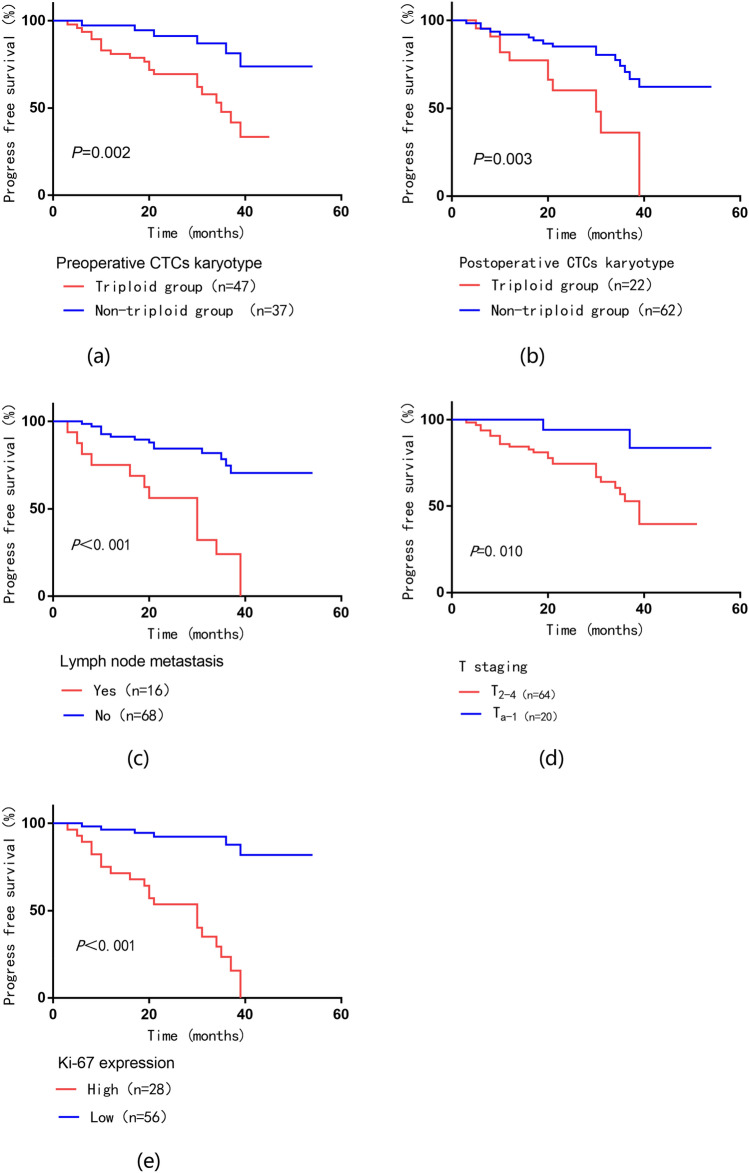
Kaplan–Meier curve analysis of the survival relationship between PFS and clinical characteristics. **a** Preoperative CTCs karyotype; **b** Postoperative CTCs karyotype; **c** Lymph node metastasis; **d** T staging; **e** Ki-67 expression

The relationship between various clinicopathological factors and PFS is shown in Table 3. Progress-free survival curves are presented by Kaplan–Meier analysis in Fig. 5. Univariate analysis showed that lymph node metastasis, T staging, Ki-67 level, preoperative CTCs karyotype, postoperative CTCs karyotype, and postoperative CTC count were significantly associated with PFS (*P* < 0.05), while age, gender, tumor diameter, and preoperative CTC count were not significant for prognosis (*P* > 0.05). In addition, in multivariate analysis, high postoperative CTC count (*P* < 0.001) and Ki-67 high expression (*P* < 0.001) were independent poor prognostic factors for PFS in bladder cancer patients.

**Table 3:** The univariate and multivariate Cox models for progression-free survival in bladder cancer patients

Parameter	Progression-free survival			
	Univariate analysis		Multivariate analysis	
	HR (95% CI)	*P*	HR(95% CI)	*P*
Age (≥ 65 vs. < 65)	0.775 (0.362–1.658)	0.511		
Gender (male vs. female)	1.145 (0.537–2.442)	0.725		
Tumor diameter (≥ 3 vs. < 3)	1.145 (0.537–2.442)	0.725		
T staging (T_2-4_ vs.T_a-1_)	5.445 (1.277–23.212)	0.022*	1.934 (0.409–9.144)	0.405
Ki-67 expression (high vs. low)	11.61 (4.533–29.733)	< 0.001*	9.268 (1.825–47.072)	< 0.001*
Lymph node metastasis (yes vs. no)	5.378 (2.514–11.505)	< 0.001*	1.958 (0.85–4.508)	0.114
Preoperative CTC count (≥ 4 vs. < 4)	2.13 (0.892–5.082)	0.089		
Postoperative CTC count (≥ 2 vs. < 2)	9.437 (3.513–25.351)	< 0.001*	4.337 (1.467–12.820)	< 0.001*
Preoperative CTCs karyotype (triploid vs. non-triploid group)	3.887 (1.556–9.715)	0.004*	0.523 (0.104–2.623)	0.431
Postoperative CTCs karyotype (triploid vs. non-triploid group)	3.157 (1.426–6.988)	0.005*	0.729 (0.30–1.776)	0.487

*Indicates *P* < 0.05

### Ki-67 levels correlated with postoperative CTC counts to further stratify PFS in patients with bladder cancer

**Fig. 6:**
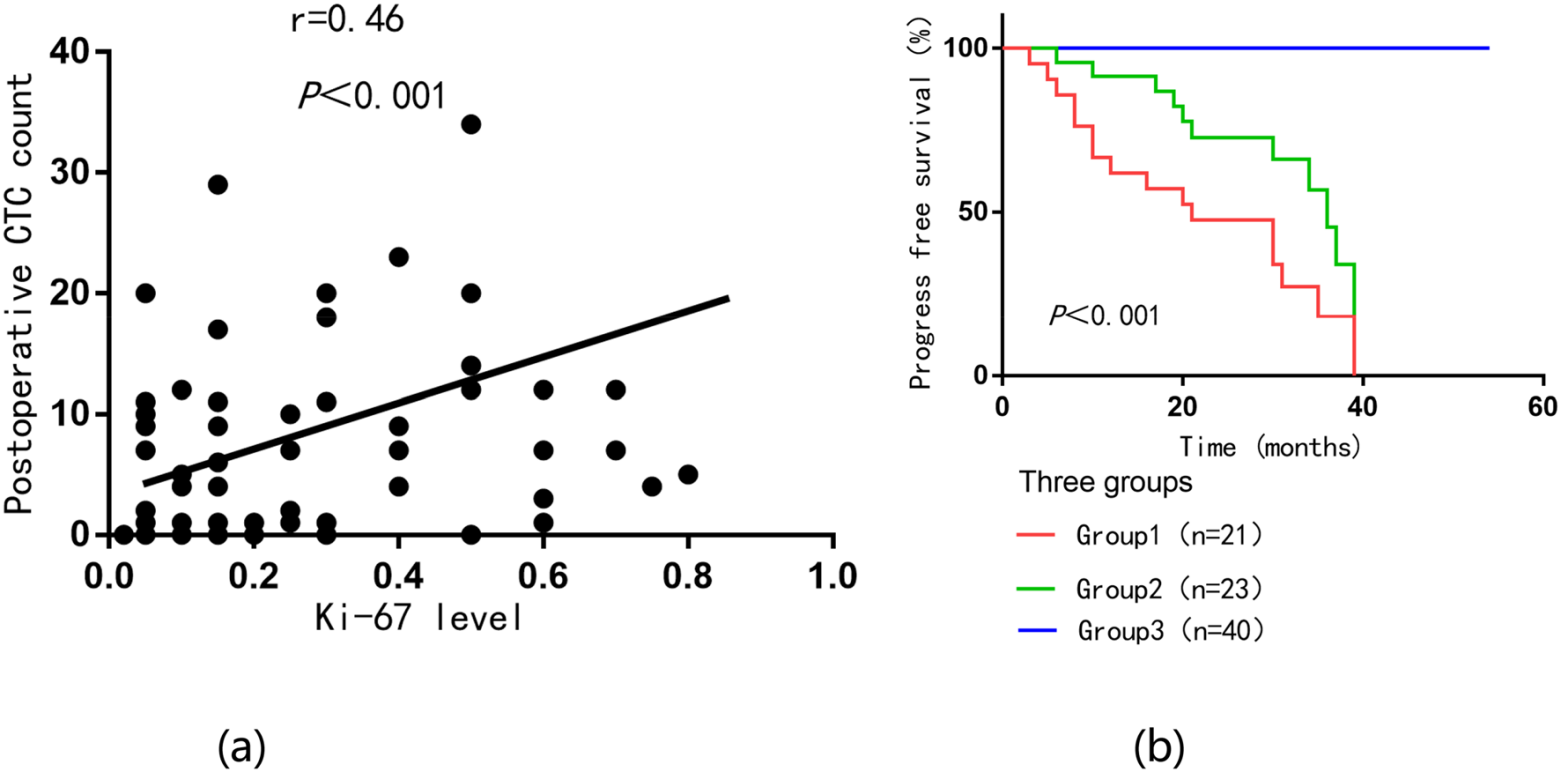
Combination of Ki-67 expression and postoperative CTC count further stratify prognosis in bladder cancer patients. **a** Correlation between Ki-67 expression and postoperative CTC count. **b** Kaplan–Meier analysis of PFS in bladder cancer patients, stratified by the Ki-67 levels and postoperative CTC counts

The potential correlation between Ki-67 levels and postoperative CTC counts was analyzed by Spearman, the result is shown in Fig. 6a. Ki-67 levels were positively correlated with postoperative CTC counts in peripheral blood (*r* = 0.46; *P* < 0.001). Since postoperative CTC count and Ki-67 level were both independent risk factors for patient prognosis (Table 3). To further risk stratify patients for PFS, as shown in Fig. 6b. Patients were divided into 3 groups: 1. Ki-67 high expression and postoperative high CTC count group; 2. Ki-67 high expression and postoperative low CTC count group or Ki-67 low expression and postoperative high CTC count group; 3. Ki-67 low expression and postoperative low CTC count group. The median PFS of patients in the Ki-67 high expression and postoperative high CTC count groups was only 20 months, and the median PFS in the Ki-67 high expression, postoperative low CTC count or Ki-67 low expression, postoperative high CTC count groups was The PFS was 27.5 months, while the median PFS of patients in the low Ki-67 expression and postoperative low CTC count group was 32 months. The log-rank method was used to test the differences in the distribution of survival time among the three groups, and the results showed that the differences in the distribution of PFS time among the three groups were statistically significant (*P* < 0.001).

## Discussion

With the continuous improvement of treatment methods, the prognosis of bladder cancer has been significantly improved, but more than 45% of patients will have tumor recurrence within 2 years, and 10% of non-muscle-invasive bladder cancer may progress to muscle-invasive bladder cancer patients [18], In addition, 50% of bladder cancer patients still have the risk of postoperative metastasis after radical resection [3]. This seriously affects the prognosis of patients, and the situation will be improved by accurately predicting the prognosis of patients, which will help to optimize the management of patients after surgery and even provide the basis for clinical treatment. Ki-67 is a correlated nuclear antigen that accurately reflects the proliferation status of cells. Ki-67 levels have been found to be closely related to the prognosis of bladder cancer [11]. However, the examination is invasive and dynamic monitoring is not possible during postoperative follow-up. Liquid biopsy is a non-invasive procedure and also allows for long-term real-time monitoring. Studies have confirmed that tumor recurrence, progression as well as metastasis are strongly correlated with the presence of CTCs [5]. The combined analysis of the two will help us to predict the prognosis of patients. The clinical value of CTCs combined with Ki-67 in predicting the prognosis of bladder cancer patients is still unreported. We evaluated for the first time the value of postoperative CTC counts combined with Ki-67 levels in accurately predicting PFS in patients with bladder cancer. In this study, the association between CTC counts and Ki-67 levels after surgery were analysed by correlation, and a positive correlation was found between them. In agreement with Song et al. [19], further survival analysis showed that patients in the high postoperative CTC count and Ki-67 high expression group had the worst prognosis, patients in the Ki-67 high expression, low postoperative CTC count or Ki-67 low expression, and high postoperative CTC count groups had an average prognosis, while patients in the low postoperative CTC count and Ki-67 low expression groups had the best prognosis, with statistically significant differences in survival analysis. Although the interaction of molecular signaling pathways between CTCs and Ki-67 remains elusive, it has been shown that the expression levels of tumor aggressiveness markers in primary tumor lesions were highly correlated with the number of CTCs [20]. The findings of this study can serve as an important complement to the currently established individualized treatment of patients with bladder cancer and tailor appropriate postoperative follow-up strategies and necessary prophylactic treatments for patients with a high risk of recurrence, for example, for patients with a high risk of recurrence, a shorter review time is recommended, which facilitates our early detection of recurrence of tumor lesions and thus timely initiation of options such as surgical treatment, bladder infusion chemotherapy, targeted therapy or local regional therapy, thus effectively improving the prognosis of patients [21]. Future studies should explore CTCs in combination with Ki-67 in clinical practice to guide personalized antitumor management.

To date, no specific biomarker has been found for predicting postoperative recurrence in patients with bladder cancer. In this evaluation, the research group also analyzed the independent risk factors affecting the postoperative PFS of patients. Multivariate analysis showed that Ki-67 and postoperative CTCs were independent predictors of poor prognosis of the disease, and Ki-67 high expression and postoperative High CTC counts were associated with shorter PFS. In recent years, there have been more studies on the prognostic significance of Ki-67 in patients with bladder cancer. Ziaran et al. [17] analyzed the relationship between immunohistochemical results and prognosis in 224 patients with bladder cancer. Survival analysis showed that Ki-67 was strongly associated with cancer-specific survival, progression-free survival, and recurrence-free survival, and in addition, ki-67 was an independent risk factor for poor patient prognosis. A meta-analysis by Tian et al. [11] similarly concluded that Ki-67 could be a valuable biomarker for the prognosis of patients with bladder cancer.

This study found that compared with preoperative CTC count, postoperative CTC count ≥ 2/7.5 ml was better in identifying high-risk patients with poor prognosis. A prospective study by Wang et al. [22] found that of 167 liver transplant patients with liver cancer, 31 of 63 patients with positive post-surgical CTCs had tumor recurrence, whereas only 23 of 104 patients with negative post-surgical CTCs had recurrence (*P* < 0.001). Multifactorial analysis suggested that positive post-surgical CTCs were an independent risk factor for tumor recurrence after liver transplantation, whereas pre-surgical. The study concluded that postoperative CTC count is a useful biomarker for predicting the risk of tumor recurrence after liver transplantation. However, Fu et al. [23] gave a different conclusion. The reasons considered were (1) residual CTCs in peripheral blood after bladder cancer surgery may be a major source of recurrence and metastasis after resection of the primary cancer and (2) due to the state of disease heterogeneity of the recruited patients (e.g. differences in treatment modalities, CTCs detection techniques, CTCs grouping criteria). In conclusion, the informative value of postoperative CTCs detection is better than that of preoperative CTCs detection, but in the future, prospective, long-term follow-up, and expanded sample size are needed to further evaluate the prognostic value of preoperative CTCs.

In addition, some studies have reported that aneuploidy karyotyping of chromosome 8 based on CTCs can better determine the prognosis and outcome of patients [5]. Lee et al. [24] demonstrated that aneuploid CTCs were strongly associated with poor prognosis in patients with colorectal cancer. Aneuploid cells could promote chromosomal instability through chromosomal missegregation events, thereby increasing the risk of tumor recurrence. Wang et al. [25] showed that the presence of triploid CTCs in the peripheral blood of patients was significantly associated with poorer progression-free survival. The present study showed that patients with triploid CTCs before or after surgery had higher postoperative tumor recurrence or mortality than those with non-triploid CTCs. The conclusions are consistent with previous reports. Therefore, triploid CTCs may belong to a specific type of CTCs as a key predictor of poor prognosis in patients [24]. By the above analysis pre-and postoperative CTCs and Ki-67 have some predictive value for the prognosis of patients with bladder cancer.

There are some limitations to this study. First, this study is a retrospective study and may have sample selection bias, such as a high proportion of advanced patients. Prospective, multicenter, and large sample studies are needed to further validate the conclusions of this study in the future. Second, this study found that the clinical value of preoperative CTCs in predicting the prognosis of bladder cancer is low and needs to be explored in-depth in the future with prolonged follow-up. Finally, this clinical study did not elaborate on the potential mechanisms associated with the correlation between CTCs and Ki-67.

In conclusion, Ki-67 high expression was associated with high postoperative CTC counts, both of which predicted a poorer prognosis for patients with bladder cancer. Post-surgical CTC counts in peripheral blood combined with Ki-67 levels could be used as a reliable indicator to predict the prognosis of patients. The combined prognostic analysis of the two could be an important complement to individualized patient care, and in the future, tailoring appropriate postoperative follow-up strategies and necessary prophylactic treatments for patients at high risk of recurrence based on the results could be advantageous and meaningful for personalized treatment of bladder cancer patients.

## Data Availability

Data used and/or analyzed in the current study are available upon reasonable request to the corresponding author.
